# Medical Record

**Published:** 1866-02

**Authors:** 


Medical Record.—We are happy to acknowledge the receipt of the first number of "The Medical Record," published by the well known firm of Wm. Wood & Co., New York. It promises to be semi-monthly; and the first number speaks well for its future success.

